# The Relationship Between Epicardial Adipose Tissue Thickness and Presence of Left Atrial Thrombus in Mitral Stenosis Patients

**DOI:** 10.21470/1678-9741-2019-0303

**Published:** 2020

**Authors:** Ender Ozal

**Affiliations:** 1 Department of Cardiology, Bagcilar Research and Education Hospital, Istanbul, Turkey.

**Keywords:** Mitral Valve Stenosis, C-Reactive Protein, Atrial Fibrilation, Thrombosis, Electrocardiography, Adipose Tissue

## Abstract

**Objective:**

To examine the relationship between the left atrial (LA) thrombus presence and the epicardial adipose tissue (EAT) thickness.

**Methods:**

Three hundred and twelve consecutive rheumatic mitral valve stenosis (RMVS) patients with mitral valve area (MVA) < 2 cm2 were included in this cross-sectional study. Patients were divided into two groups, those with and those without LA thrombus. Routine biochemical analysis and electrocardiographic examinations were carried out. EAT was measured using transthoracic echocardiography.

**Results:**

LA thrombus was determined in 84 (26.9%) RMVS patients. In echocardiographic examinations, higher mean gradient and LA diameter as well as lower MVA were found in the group with LA thrombus (*P*<0.001). In this group, higher C-reactive protein (CRP) and EAT values were also determined (*P*<0.001). There was significant correlation between EAT and MVA, CRP, LA appendage peak flow velocity, LA anteroposterior diameter, and mean gradient (*P*<0.001). Higher EAT values were identified as independently associated with the presence of LA thrombus (odds ratio 59.5; 95% confidence interval 12.1-290.10; *P*<0.001).

**Conclusion:**

Transthoracic echocardiography, routinely used in patients with RMVS, can measure EAT to determine patients who are under risk for thrombus.

**Table:** 

Abbreviations, acronyms & symbols				
2D	= Two-dimensional		OR	= Odds ratio
AF	= Atrial fibrillation		PAF	= Paroxysmal atrial fibrillation
BMI	= Body mass index		RAAS	= Renin-angiotensin-aldosterone system
CAD	= Coronary artery disease		SPSS	= Lactate dehydrogenase
CV	= Cardioversion		TEE	= Transesophageal echocardiography
DM	= Creatine kinase-muscle/brain		TSH	= Thyroid-stimulating hormone
ECG	= Electrocardiography		TSH	= Thyroid-stimulating hormone
ECG	= Electrocardiography		VitD	= Vitamin D
HT	= Hypertension			

## INTRODUCTION

Rheumatic heart diseases are a significant cause of morbidity and mortality in undeveloped and developing countries^[1]^. Thrombus occurring in the left atrium (LA) in mitral stenosis (MS) patients leads to embolic complications, of which ischemic stroke is the most serious one^[2]^. Blood stasis developing within LA due to mitral valve stenosis cannot explain the formation of thrombus alone. There is significant data available showing the relationship between two of the building blocks of thrombus formation, hypercoagulability and inflammation. The inflammatory process continues subclinically in rheumatic heart diseases.

Epicardial adipose tissue (EAT) is a metabolically active endocrine organ which secretes fatty acids, adiponectin, inflammatory cytokines, and prothrombotic factors^[3-6]^. EAT is clinically related to abdominal visceral adiposity^[7]^, coronary artery disease (CAD)^[8]^, subclinical atherosclerosis^[9]^, and cardiac morphology^[10]^. Recently, a relationship between thrombus occurrence in peripheral circulation and EAT has been determined^[11]^. However, there is no data regarding the relationship between EAT and the presence of LA thrombus, which is responsible for 10-20% of all strokes. EAT, linked to many cardiovascular diseases through inflammatory activity, may also be related to intracardiac thrombus formation^[7-10]^. In addition to EAT thickness being easy and noninvasive to measure with transthoracic echocardiography (TTE), its reliability is correlated with the gold standard method of magnetic resonance imaging (MRI)^[12]^. In our study, we aimed to investigate the relationship between LA thrombus presence and EAT in patients with rheumatic mitral valve stenosis (RMVS).

## METHODS

### Study Population

RMVS patients with mitral valve area (MVA) < 2 cm^2^ have been included in this cross-sectional study consecutively. All patients underwent TTE and transesophageal echocardiography (TEE) and were divided into two groups according to the presence of LA thrombus. The exclusion criterion for the study was the presence of heart failure, acute coronary syndrome, previous cardiac surgery, renal impairment (estimated glomerular filtration rate [eGFR] < 60 ml/min/1.73 m^2^), abnormal liver function (elevation of transaminase levels of more than three times the upper limit of normal), active infectious or inflammatory diseases, connective tissue disease, smoking, malignancy, thyroid disease, and other hematological diseases. The medical history, physical examination, and biochemical analysis of all patients were recorded. Atrial fibrillation (AF) presence was identified through 12-lead electrocardiography. Height and body weight were measured to calculate body mass index (BMI). Hypertension (HT) was defined as systolic blood pressure ≥ 140 mmHg and/or diastolic blood pressure ≥ 90 mmHg or medication use. Diabetes mellitus (DM) was defined as fasting blood glucose level ≥ 126 mg/dl or use of insulin or an oral hypoglycemic medication. CAD was assessed from patients’ medical reports. Non-smokers were defined as patients who had never smoked or chewed tobacco in any form.

Informed consent was obtained from all patients. This study obeys the Declaration of Helsinki and the trial approved by the local ethics committee.

### Laboratory Evaluation

Blood was taken after 12 hours fasting, in the morning, between 8 and 9 am. Serum parameters analyzed were creatinine, blood glucose, total cholesterol (TC), low-density lipoprotein cholesterol (LDL-C), high-density lipoprotein cholesterol (HDL-C), and triglyceride (TG). C-reactive protein (CRP) levels were measured using the nephelometric method (Beckman Coulter IMMAGE 800).

### Echocardiographic Assessment

Two-dimensonal TTE was performed using a 4 MHz, sector-type transducer probe for each subject (Vivid 7, GE Medical, USA). All patients were kept in the left lateral decubitus position based on the recommendations by the American and European Societies of Echocardiography guideliness^[13]^. The modified Simpson method with two-dimensional echocardiographic apical 4-chamber view was used to calculate the left ventricular ejection fraction (LVEF). The MVA was measured using the planimetric method. TEE was performed using a commercially avaliable machine (Vivid 7, GE Medical, USA), with a mutiplane TEE probe and a 3,5 MHz phased array transducer. After the patients had been administered pharyngeal topical anaesthesia with lidocaine spray, the probe advanced a depth of 25-35 cm and obtained optimum imaging. The images were evaluated for the presence or absence of thrombus in the LA and left atrial appendage (LAA). LAA clot was diagnosed by the presence of clearly deﬁned echogenic intracavitary mass with an echo texture different from that of the underlying endocardium and not due to the pectinate muscle. All images were archived and evaluated by two independent echocardiographer cardiologists and any discrepancy was resolved by consensus. Artefact images were required at different TEE rotational angles. With 50 randomly selected patient images, the intra and interobserver variabilities in terms of LA thrombus were evaluated and determined as 2.9% and 3.8%, respectively.

The maximum EAT was measured at the point on the free wall of the right ventricle at end-diastole, perpendicular to the aortic annulus for the parasternal long-axis view. Epicardial fat was defined as the relatively echo-free space between the outer wall of the myocardium and the visceral layer of the pericardium. EAT was defined as the average of three cardiac cycles from each echocardiographic view. In 30 randomly selected subjects, EAT was re-measured by echocardiologists from the same pre-selected images using the same method of measurement as the first iteration. The coefficients of intraobserver and interobserver variations were 4.7% and 7.3%, respectively.

### Statistical Analysis

The Statistical Package for the Social Sciences (SPSS) for Windows (SPSS 17.0, Chicago, Illinois, USA) software package, version 17.0, was used in all analyses. The continuous variables were expressed as mean ± standard deviation (SD) (for parameters with normal distribution) and median interquartile range (IQR) (for parameters without normal distribution), and categorical variables were expressed as percentages. The chi-square test was used to compare categorical variables between the groups. Analysis of normality was performed with the Kolmogorov-Smirnov test. The independent samples *t*-test was used to compare continuous variables with normal distribution and the Mann-Whitney U test was used to compare continuous variables without normal distribution. Association between EAT and other continuous variables was assessed with Spearman and Pearson correlation coefficients. Variables with a *P*-value < 0.25 in univariate analysis were incorporated in the multivariate model. Multivariate logistic regression analysis was performed to identify independently associated factors with the presence of LA thrombus. In order to determine the optimal cutoff value for EAT thickness in terms of development of LA thrombus, receiver operating characteristic (ROC) curve analysis was used. A two-sided *P*-value < 0.05 was considered significant within a 95% confidence interval (CI).

## RESULTS

The total number of patients was 312, with LA thrombus determined in 84 (26.9%) and not determined in 228 (73.1%) of them. The mean age of the group with LA thrombus was 54.5±9.1 years and it was consisted of 56 (66.3%) women. No significant differences in terms of age, gender, BMI, and comorbid conditions (DM, HT, and CAD) were found between the groups. A higher incidence of AF was determined in the LA thrombus group (Table 1).

**Table 1:** Comparison between clinical and laboratory findings.

Variables	LAT (+) n=84	LAT (-) n=228	*P*-value
Age (years)	54.5±9.1	53.5±12.9	0.529
Females	56 (66.7%)	164 (71.9%)	0.241
BMI (kg/m^2^)	25.2±1.4	25.4±1.5	0.331
AF	36 (42.9%)	27 (11.8%)	<0.001
CAD	9 (10.7%)	26 (11.4%)	0.522
DM	9 (10.7%)	24 (10.5 %)	0.553
Hypertension	13 (15.5%)	36 (15.8%)	0.550
Stroke	5 (6%)	6 (2.6%)	0.158
Mean gradient (mmHg)	12.2 (4.17)	10.3 (4.18)	<0.001
Mitral valve area (cm^2^)	0.9±0.1	1.1±0.2	<0.001
LVEF (%)	59.0±3.7	59.9±4.2	0.08
LAAPV (cm/s)	25.4±4.8	36±10.3	<0.001
LAAPD (mm)	46.9±7.6	40.9±6.7	<0.001
Glucose (mg/dl)	82.1±11.4	80.4±11.6	0.264
LDL-C (mg/dl)	128.0 (45.7)	122.6 (31.7)	0.212
CRP (mg/L)	4.7 (2.8)	2.8 (1.7)	<0.001
eGFR(ml/min/1.73m^2^)	84.8±8.9	84.9±8.6	0.941
EAT thickness (mm)	4.7±0.7	3.11±0.6	<0.001

*P*<0.05 was considered statistically significant. Data are presented as mean±standard deviation, median (interquartile range), and frequency (percentages). AF=atrial fibrillation; BMI=body mass index; CAD=coronary artery disease; CRP=C-reactive protein; DM=diabetes mellitus; EAT=epicardial adipose tissue; eGFR=estimated glomerular filtration rate; LAAPD=left atrial anteroposterior diameter; LAAPV=left atrial appendage peak flow velocity; LAT=left atrial thrombus; LDL-C=low-density lipoprotein cholesterol; LVEF=left ventricular ejection fraction

When the laboratory results were analyzed, a difference between the two groups in terms of glucose, LDL-C, and eGFR was not determined. The group with LA thrombus had higher CRP levels (Table 1). Analyzing the echocardiographic parameters, while a difference could not be determined between the groups in terms of LVEF, in the LA thrombus group the left atrial anteroposterior diameter (LAAPD) and mitral valve gradient values were higher, and the MVA and left atrial appendage peak flow velocity (LAAPV) were found to be lower (Table 1). Also, EAT thickness was higher in those with AF than in those without it (3.91 mm *vs*. 3.46 mm, respectively, *P*=0.002)

As a result of correlation analysis, a significant level of correlation was determined between EAT thickness and LAAPV, LAAPD, mean gradient, MVA, and CRP (*P*<0.001) (Table 2). The results of the univariate analysis are shown in Table 3.

**Table 2:** Univariate correlates of the epicardial adipose tissue thickness in the study population.

Variables	r	*P*-value
Age (years)	-0.018	0.745
Body mass index (kg/m^2^)	0.024	0.679
LVEF (%)	-0.072	0.204
LAAPV (cm/s)	-0.350	<0.001
LAAPD (mm)	0.257	<0.001
Mean gradient (mmHg)	0.242	<0.001
Mitral valve area (cm^2^)	-0.235	<0.001
Glucose (mg/dl)	0.034	0.550
eGFR (ml/min/1.73m^2^)	0.021	0.718
LDL-C (mg/dl)	0.017	0.771
CRP (mg/L)	0.323	<0.001

*P*<0.05 was considered statistically significant. CRP=C-reactive protein; eGFR=estimated glomerular filtration rate; LAAPD=left atrial anteroposterior diameter; LAAPV=left atrial appendage peak flow velocity; LDL-C=low-density lipoprotein cholesterol; LVEF=left ventricular ejection fraction

**Table 3:** Factors related to left atrial thrombus according to univariate regression analysis.

Variables	Odds ratio (95% confidence ınterval)	*P*-value
LAAPD	1.12 (1.07-1.16)	<0.001
LAAPV	0.848 (0.810-0.887)	<0.001
EAT thickness	24.76 (11.85-51.7)	<0.001
MVA	0.10 (0.03-0.32)	<0.001
CRP	1.53 (1.33-1.77)	<0.001
AF	5.5 (3.0-10.0)	<0.001

*P*<0.05 was considered statistically significant. AF=atrial fibrillation; CRP=C-reactive protein; EAT=epicardial adipose tissue; LAAPD=left atrial anteroposterior diameter; LAAPV=left atrial appendage peak flow velocity; MVA=mitral valve area

In the multivariate logistic regression analysis, it has been determined that there is an independent association between the existence of LA thrombus and LAAPV, LAAPD, CRP, EAT thickness, and the existence of AF (Table 4).

**Table 4:** Factors related to left atrial thrombus according to multivariate logistic regression analysis.

Variables	Odds ratio (95% confidence ınterval)	*P*-value
LAAPD	1.131 (1.034-1.248)	0.008
LAAPV	0.736 (0.635-0.853)	<0.001
EAT thickness	43.968 (11.6-165.5)	<0.001
MVA	0.596 (0.037-9.525)	0.714
CRP	2.535 (1.529-4.205)	<0.001
AF	15.853 (2.650-94.836)	0.002

*P*<0.05 was considered statistically significant. AF=atrial fibrillation; CRP=C-reactive protein; EAT=epicardial adipose tissue; LAAPD=left atrial anteroposterior diameter; LAAPV=left atrial appendage peak flow velocity; MVA=mitral valve area

**Fig. 1:**
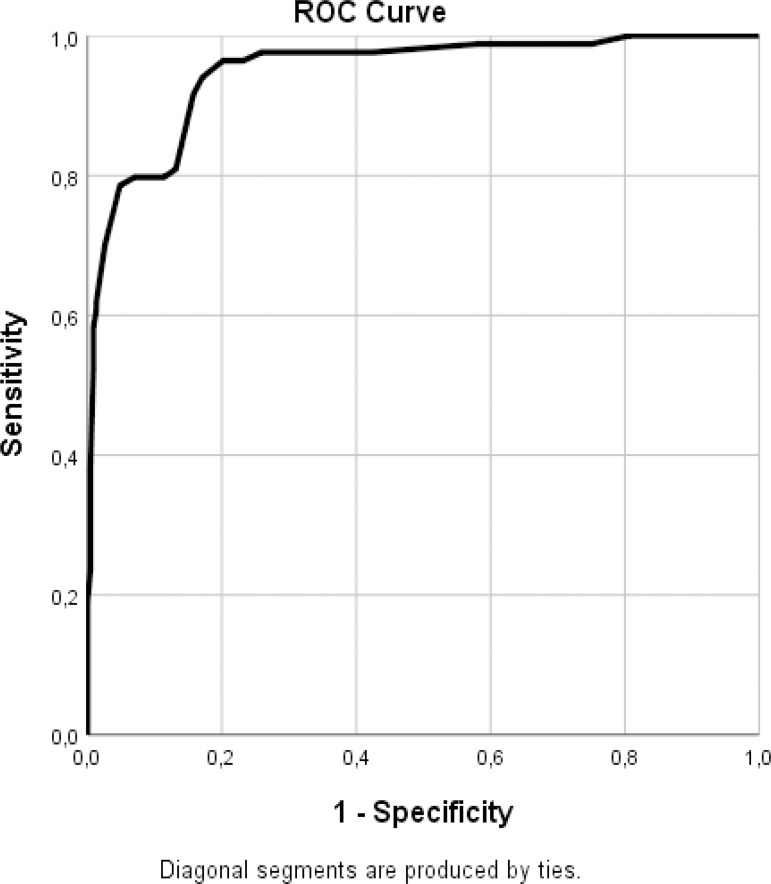
Receiver operating characteristic (ROC) curve for the epicardial adipose tissue thickness in predicting the development of left atrial thrombus.

ROC analysis provided a cutoff value of 4.05 for EAT thickness to predict LA thrombus with 79% sensitivity and 89% specificity (area under the curve 0.94; 95% CI 0.92-0.97) (Figure 1).

## DISCUSSION

In our study, thicker epicardial fat values were determined in patients with LA thrombus. This relationship continued independently of important factors in thrombus formation, such as the presence of AF, the LA diameter, and the degree of MS.

Epicardial fat is the adipose tissue accumulated between the visceral pericardium and the myocardium. The locations of the most parts of epicardial fat tissue are, in order, right ventricular free wall, left ventricular free wall, the surroundings of the atriums, and the adventitia of the coronary artery branches, from the epicardial surface towards the myocardium^[14]^. It has been shown that EAT plays a role in the pathophysiology of artherothrombosis through the proinflammatory cytokines and prothromboic factors it secretes^[15-18]^. Through inflammatory cytokines and thrombotic factors, such as interleukin-6, interleukin-1ß, monocyte chemotacticprotein-1 (MCP-1), tumor necrosis factor-alpha, visfatin, and leptin, they create a prothrombotic surface by impacting the endothelium and the endocardiac morphology, as systemic and paracrine. Goldsmith et al. have shown the contribution of LA endocardial thickening, endothelium loss, and prothrombotic changes in the endothelium on the formation of intra-atrial thrombus in MS patients^[19]^. RMVS, a late finding of rheumatic carditis, is an inflammatory and autoimmune disease, and it has been determined that the inflammatory process continues subclinically^[4,5]^. There is important data regarding the relationship between inflammation and prothrombotic situation^[20,21]^. Currently, LA dilation, myocardial remodelling, and haemodynamic changes are important mechanisms in LA thrombus, however they may not explain thrombus formation alone^[22,23]^. Higher CRP levels have been determined in the rheumatic valve patients in comparison to the control group, and in those with LA spontaneous echo contrast compared to those that do not have it. In addition to the determination of a positive correlation between EAT thickness and CRP in our study, we determined, for the first time, higher CRP levels in those with LA thrombus than in those without it. These results may reflect the close relationship between thicker EAT and inflammation, as well as with systemic prothombotic state.

The Framingham Heart Study, one of the most comprehensive prospective studies, has shown a close relationship between cardiovascular diseases and cancer and EAT in the five-year follow-up of 3,000 patients. Studies have shown that there is a positive correlation between coronary artery calcification, coronary and carotid artherosclerosis, and EAT^[24]^. It has also been determined that there is a relationship between periatrial fat and AF development independently of important factors, such as HT, DM, and LA diameter. The inflammatory cytokines secreted by periatrial fat has caused the formation of atrial substrate for AF on the LA structure^[5]^. Although periatrial fat was not measured in our study, we determined a link between epicardial fat thickness and the formation of LA thrombus, as well as anatomic features, such as MVA and LAAPD, and functional features, such as LAAPV and mean gradient. Mahabadi et al.^[25]^ have determined a relationship between EAT and AF frequency, as well as LA diameter, in the AF patients that they’ve examined with non-contrast computed tomography. In previous studies, an increasing amount of EAT was reported to be associated with the presence of AF. EAT, a unique fat deposit, was directly contiguous with atrial and ventricular myocardium and is highly metabolically active. We also found a close association between EAT and AF in our study. Probably, EAT serving as an abundant source of inflammatory mediators may predispose patients to AF by increasing the local inflammatory burden, which directly damaged the atrium. This may show that EAT may contribute to thrombus formation with local effect in addition to systemic cardiometabolic risk factors. Despite the fact that there are no studies to date regarding the relationship between intracardiac thrombus formation and EAT, a close relationship between thrombus in peripheral circulation and thicker EAT has been determined by Mazzoccoli et al.^[11]^.

EAT thickness measurements can be carried out easily and noninvasively with TTE. During TTE application, frequently used in clinical practice with rheumatic heart patients, with the assessment of EAT thickness, patients at high risk of thrombus can be determined. When the close relationship between EAT thickness and cardiovascular diseases is considered, it may be an important parameter in terms of risk stratification and follow-up. EAT is a component of visceral adipose tissue, and it has been shown that visceral adipose tissue decreases with weight loss. As the relationship between EAT and the incidence of cardiometabolic risk factors has been shown in various patient groups, EAT being modified through diet and lifestyle changes can lead to positive results in terms of artherosclerosis, inflammation, and prothrombotic process.

Echocardiographic EAT measurement is an inexpensive, noninvasive, reproducible, and direct measure of visceral fat. And TEE may have an important role in predicting high risk for stroke and stratifying cardiovascular risk in both clinical care and research setting.

### Limitation

Our study has some limitations. We measured EAT thickness, rather than volume. Because echocardiography measures EAT thickness linearly, echocardiographic EAT thickness may not reflect the total epicardial fat volume exactly. However, despite MRI being the gold standard in terms of EAT measurement, no clinical difference was determined in terms of TTE measurement^[16]^. Measurement of EAT thickness with TTE is easier, cheaper, and less time-consuming compared to the labor-intensive work for measurements of volumetric epicardial fat, thus it is more suitable for use in daily clinical practice. The inflammatory cytokines, whose relationship with EAT has previously been determined, could not be measured.

## CONCLUSION

In our study, we detected thicker epicardial adipose values in RMVS patients with LA thrombus. This relationship continued independently of the severity of MS and the existence of AF. The echocardiographic assessment of epicardial fat may also have the potential to be a simple and reliable marker of LA thrombus and increased emboli risk. Because echocardiography is likely to be routinely performed in RMVS patients, it can allow us to manage patients with tendency to thrombus without additional applications and costs.

**Table:** 

Author's roles & responsibilities	
EO	Substantial contributions to the conception or design of the work; or the acquisition, analysis, or interpretation of data for the work; drafting the work or revising it critically for important intellectual content; agreement to be accountable for all aspects of the work in ensuring that questions related to the accuracy or integrity of any part of the work are appropriately investigated and resolved; final approval of the version to be published
